# Seroprevalence of 13 common pathogens in a rapidly growing U.S. minority population: Mexican Americans from San Antonio, TX

**DOI:** 10.1186/1756-0500-4-433

**Published:** 2011-10-21

**Authors:** Rohina Rubicz, Charles T Leach, Ellen Kraig, Nikhil V Dhurandhar, Barry Grubbs, John Blangero, Robert Yolken, Harald HH Göring

**Affiliations:** 1 Department of Genetics, Texas Biomedical Research Institute, PO Box 760549, San Antonio, TX 78245-0549, USA; 2 Department of Pediatrics, University of Texas Health Science Center at San Antonio, 7703 Floyd Curl Drive, San Antonio, TX 78229, USA; 3 Department of Cellular and Structural Biology, University of Texas Health Science Center at San Antonio, 7703 Floyd Curl Drive, San Antonio, TX 78229, USA; 4 Infections and Obesity Laboratory, Pennington Biomedical Research Center, Louisiana State University System, 6400 Perkins Road, Baton Rouge, LA 70808-4124, USA; 5 The Stanley Division of Developmental Neurovirology, Johns Hopkins School of Medicine, 600 N. Wolfe Street, Baltimore, MD 21287-4933, USA

## Abstract

**Background:**

Infection risks vary among individuals and between populations. Here we present information on the seroprevalence of 13 common infectious agents in a San Antonio-based sample of Mexican Americans. Mexican Americans represent the largest and most rapidly growing minority population in the U.S., and they are also considered a health disparities population.

**Methods:**

We analyzed 1227 individuals for antibody titer to *Chlamydophila pneumoniae, Helicobacter pylori, Toxoplasma gondii*, cytomegalovirus, Epstein-Barr virus, herpes simplex virus-1, herpes simplex virus-2 (HSV-2), human herpesvirus-6 (HHV-6), varicella zoster virus (VZV), adenovirus-36, hepatitis A virus, and influenza A and B. Seroprevalence was examined as a function of sex, age, household income, and education.

**Results:**

Seroprevalence estimates ranged from 9% for *T. gondii* to 92% for VZV, and were similar in both sexes except for HSV-2, which was more prevalent in women. Many pathogens exhibited a significant seroprevalence change over the examined age range (15-94 years), with 7 pathogens increasing and HHV-6 decreasing with age. Socioeconomic status significantly correlated with serostatus for some pathogens.

**Conclusions:**

Our findings demonstrate substantial seroprevalence rates of these common infections in this sample of Mexican Americans from San Antonio, Texas that suffers from high rates of chronic diseases including obesity and type-2 diabetes.

## Background

The presence of antibodies specific to a given pathogen is indicative of a current or previous exposure to an infectious agent either through infection or vaccination. Hence, seroprevalence is an often-used measure of the frequency of common infections in a population. Seroprevalence for many agents is noted to increase with age, most likely due to increased opportunity for exposure over the course of an individual's lifetime. In some instances, such as for herpes simplex virus type 2 in the general U.S. population, seroprevalence is unequal between the sexes [1], but more often there is no sex bias. Other factors, including socioeconomic status, household crowding, breastfeeding practices, food-production practices, and level of parental education, have also been shown to influence the seroprevalence of particular pathogens within a population [2-6]. Thus, prevalence of exposure to a particular pathogen, as reflected in seroreactivity, tends to vary between and within populations and is often associated with disparities in socioeconomic status and differences in ethnic background [2,7,8].

There is a growing body of evidence suggesting that inflammation associated with persistent infection may contribute to the development of cardiovascular disease and other chronic diseases of aging [9-11]. In certain cases, particular pathogens have been linked to chronic disease. For example, infection with *Chlamydophila pneumoniae* (formerly *Chlamydia pneumoniae*) has been found to be associated with atherosclerosis, coronary heart disease, myocardial infarction, and general risk of cardiovascular mortality [12,13]. Other pathogens, including *Helicobacter pylori*, cytomegalovirus, Epstein-Barr virus, hepatitis A virus, and herpes simplex 1 virus have also been identified as potentially contributing to cardiovascular disease, although there are discrepancies between studies [14-17]. It has also been speculated that pathogen burden (i.e., the number of different infections in an individual) may contribute to atherosclerosis or other chronic disease. Studies have shown an association between pathogen burden and C-reactive protein levels, clinical disease outcomes, and overall risk of cardiovascular disease [18-21].

Mexican Americans have been shown, in general, to have unequal access to health care and to suffer greater morbidity and mortality due to a variety of both infectious and chronic diseases, including heart disease and diabetes, in comparison with the overall U.S. population [22]. Focusing on this "health disparities population" is even more crucial because Hispanics are the largest and fastest growing minority population in the U.S., surpassing 45 million individuals and comprising 15% of the total population, and Mexican Americans constitute the majority (> 60%) of this group [23].

We have here specifically investigated Mexican Americans from around San Antonio, Texas, which may not be representative of all US-based Hispanics. As some of the relatives of study participants resided in Monterrey, Nuevo León, Mexico, we compare the seroprevalence rates between the two locations (U.S. versus Mexico). We report the seroprevalence, as a function of sex, age, household income, and education level, in this sample of Mexican Americans for thirteen relatively common pathogens, which were chosen based on three criteria: i) reported moderate to high frequency in the general U.S. population, ii) relative ease of transmission (i.e., thought to be primarily acquired through casual person-to-person contact or through respiratory secretions, at least for most of the investigated pathogens), and, iii) suspected involvement in cardiovascular disease or other chronic disorders. Typical infections with most of these agents cause subclinical or relatively mild symptoms, although some can cause severe manifestations.

## Methods

### Participants

Individuals participating in this study included 1,227 members of randomly ascertained, extended Mexican American families from San Antonio, TX, and the surrounding region [24] (Table 1). The subjects were recruited during the years 1991-1995 for participation in the San Antonio Family Heart Study (SAFHS), which seeks to identify genetic risk factors for cardiovascular disease. In addition, another 147 individuals residing in Monterrey, Nuevo León, Mexico, were recruited to participate in the study because they are family members of participants from San Antonio, and they are required to build the multigenerational genealogies of the study participants. Participants range in age from 15 to 95 years. Study protocols were approved by The University of Texas Health Science Center at San Antonio and all participants signed statements of informed consent. Many types of data were collected, including information on sex, age, education level, household income, and location of residence.

**Table 1:** Participant characteristics

Characteristic	Value (percent)
Sex	
Male	482 (39.3%)
Female	745 (60.7%)
Age	
Age range	15-94 years
Mean age	39 years
Education*	
Elementary school (1-8 years)	329 (27.9%)
Some high school (9-11 years)	324 (27.5%)
High school graduation (12 years)	370 (31.4%)
Some college (> 12 years)	157 (13.3%)
Monthly Household Income†	
Under $500	181 (16.2%)
$500-$749	159 (14.2%)
$750-$999	143 (12.8%)
$1000-$1249	171 (15.3%)
$1250-$1499	117 (10.5%)
$1500-$1999	123 (11.0%)
$2000-$2999	127 (11.4%)
$3000 and above	95 (8.5%)
Residence	
Texas	1211(98.7%)
Other US location	16 (1.3%)

*Information available for 1180 participants

†Information available for 1116 participants

### Serology

Following an overnight fast, blood samples were collected from participants using EDTA vacutainers at the time of recruitment (1991-1995). At that time, frozen plasma aliquots were obtained as previously described [25] and stored at -80°C. These archived samples were thawed just prior to use for antibody determinations, which were performed during 2008-2010. Of the infectious pathogens examined in this study, two are bacterial, *C. pneumoniae* and *H. pylori;* one, *Toxoplasma gondii,* is a protozoan; and the remaining pathogens are viruses. The viral pathogens include six members of the herpes virus family: cytomegalovirus (CMV); Epstein-Barr virus (EBV); herpes simplex type I virus (HSV-1); herpes simplex type II virus (HSV-2); human herpesvirus 6 (HHV-6); and varicella zoster virus (VZV). The remaining viruses are adenovirus 36 (Ad-36); hepatitis A virus (HAV); influenza A virus; and influenza B virus. All of these pathogens typically induce measureable humoral responses in exposed immune competent individuals. Although vaccinations may affect pathogen seroprevalence rates, in this study population immunizations were only available for influenza A and B when these samples were collected, so all other seroprevalence rates directly reflect current or prior exposure to the relevant pathogens.

Commercially available ELISA kits were used to determine immunoglobulin G (IgG) antibody titers to: *C. pneumoniae *(Bioclone Australia Pty Ltd., Marrickville NSW Australia); *H. pylori *and CMV (Inverness Medical Professional Diagnostics, Palatine, IL); *T. gondii*, EBV (specifically to EBV nuclear antigen [EBNA]), VZV, influenza A, and influenza B (IBL America, Minneapolis, MN); HSV-1 and HSV-2 (Focus Diagnostics Inc., Philadelphia, PA); HAV (Bio-Rad Laboratories, Redmond, WA); and HHV-6 (Advanced Biotechnologies, Rockville, MD). A published serum neutralization test was utilized for measuring Ad-36 antibodies [26].

For commercial ELISA assays, the manufacturers' instructions were used to determine seropositive/seronegative status according to the following absorbance values: seronegative if ≤ 0.9; indeterminate if > 0.9 and < 1.1; and seropositive if ≥1.1. The cut-off value for *T. gondii *seropositivity was equivalent to 10 IU/ml. Ad-36 analyses were run in duplicate, with specimens assigned as seropositive if both replicates had neutralization titres ≥1:8, otherwise they were considered to be seronegative.

### Statistical analysis

Because this study included relatives, we used a variance components (VC) model implemented in a software package, SOLAR [27], that is explicitly designed for analysis of pedigree data. The non-independence of relatives due to the additive effects of genes in aggregate was properly taken into account using a random effects kinship parameter, which absorbs phenotypic (co-)variation due to the expected overall genetic similarity among relatives [28]. For analysis of dichotomous phenotypes (such as seropositive versus seronegative status), a liability threshold model (i.e., a theoretical and unobservable quantitative liability was assumed to underlie the dichotomous phenotype, with those individuals exceeding a threshold value considered to be affected) was used within the VC framework [29]. The effects of sex, age, household income, education, and location (U.S. or Mexico) on serostatus of the pathogens were assessed by maximum likelihood methods using a fixed effect linear regression model within the mixed model framework. Those variables not of primary interest in an analysis were treated as nuisance parameters, including sex, age, age^2^, sex by age, and sex by age^2 ^(for assessing the effects of income, education, and location on serostatus); age and age^2 ^(for assessing sex effects); and sex (for assessing effects due to age). The resulting p-values were adjusted for multiple testing (i.e., pathogens investigated) using the Sidàk approximation [30].

## Results

This study is based on 1,227 Mexican American individuals taking part in the San Antonio Family Heart Study (SAFHS) [24]. They are members of extended families that were ascertained irrespective of any disease status. An overview of the relevant characteristics of the study participants is given in Table 1. The SAFHS also includes another 147 individuals living in Mexico (largely in Monterrey, Nuevo León, approximately a five hour drive by car from San Antonio), who were recruited to participate in the study because they are relatives of study participants from San Antonio. As the first step in our analyses, we determined whether there were differences in pathogen seroprevalence estimates between the 1,227 individuals living in the U.S. (Table 2) and the 147 participants from Mexico (Additional File 1: Table S1). Seroprevalence rates for six of the 13 pathogens exhibited significant differences in sex- and age-adjusted seroprevalence by location, after correcting for multiple testing (i.e., examined pathogens) (Table 3). For example, HAV seroprevalence was higher in Mexico (p = 2.3 × 10^-7^), while influenza B seroprevalence was significantly lower (p = 4.5 × 10^-5^). As the sample from Mexico is relatively small and many factors may contribute to different seroprevalence rates between both countries, we decided to exclude individuals from Mexico from subsequent analyses, which focused only on the 1, 227 participants living in the U.S.

**Table 2:** Seroprevalence by sex and age

Sex and Age Categories	count	*Cp*	*Hp*	*Tg *	CMV	EBV	HSV-1	HSV-2	HHV-6	VZV	Ad- 36	HAV	IA	IB
Females														
< 20	71	84.5	31.0	1.4	26.8	40.8	80.3	7.0	93.0	95.8	14.1	32.4	77.5	60.6
20-29	188	85.1	49.5	8.5	38.3	46.3	78.2	17.6	87.8	92.6	12.8	63.3	71.3	51.6
30-39	149	83.9	53.7	6.0	63.8	47.0	83.2	21.5	81.9	91.9	13.4	78.5	69.1	56.4
40-49	165	81.2	60.0	6.1	72.1	42.4	82.4	32.1	75.8	88.5	10.9	81.2	73.9	52.7
50-59	86	93.0	72.1	11.6	76.7	47.7	86.0	34.9	74.4	91.9	16.3	95.3	69.8	46.5
60-69	49	77.6	79.6	16.3	75.5	36.7	83.7	44.9	67.3	85.7	20.4	98.0	85.7	69.4
≥70	37	81.1	81.1	27.0	91.9	35.1	89.2	37.8	64.9	97.3	18.9	86.5	89.2	59.5
subtotal	745	84.2	57.0	8.6	59.3	44.0	82.1	25.4	80.4	91.5	13.8	74.5	73.7	54.6
Males														
< 20	75	88.0	32.0	4.0	28.0	45.3	69.3	5.3	90.7	94.7	10.7	29.3	81.3	62.7
20-29	122	81.1	45.9	2.5	32.8	54.1	78.7	13.1	93.4	92.6	17.2	59.8	78.7	62.3
30-39	85	84.7	67.1	10.6	52.9	47.1	82.4	21.2	84.7	88.2	14.1	78.8	80.0	68.2
40-49	89	92.1	67.4	9.0	61.8	56.2	87.6	13.5	75.3	95.5	14.6	86.5	80.9	62.9
50-59	56	89.3	67.9	19.6	64.3	35.7	85.7	16.1	78.6	91.1	5.4	91.1	83.9	44.6
60-69	29	100	65.5	17.2	75.9	48.3	82.8	27.6	72.4	100	13.8	96.6	86.2	58.6
≥70	26	100	84.6	38.5	73.1	65.4	88.5	38.5	61.5	96.2	3.8	92.3	84.6	76.9
subtotal	482	88.0	57.3	10.2	49.4	50.0	81.1	16.0	83.4	93.2	12.9	71.0	81.1	62.0

Overall (counting indeterminates as seropositives)	1227	85.7 (91.2)	57.1 (61.3)	9.2 (12.0)	55.4 (61.8)	46.4 (69.8)	81.7 (83.2)	21.7 (22.9)	81.6 (88.9)	92.2 (98.1)	13.4 (13.4)	73.1 (73.2)	76.6 (89.2)	57.5 (76.5)

*Cp = C. pneumoniae; Hp = H. pylori; Tg = T. gondii*; IA = Influenza A; IB = Influenza B

**Table 3:** Predictors of serostatus

Pathogen	Location regression coefficient* (*p*-value)	Sex regression coefficient† (*p*-value)	Age regression coefficient (*p*-value)	Education regression coefficient (*p*-value)	Income regression coefficient (*p*-value)
*C. pneumoniae*	**-0.919 (6.72 × 10^-4^)**	0.242 (0.023)	-0.007 (0.021)	0.108 (0.095)	0.066 (0.238)
*H. pylori*	-0.032 (0.832)	0.057 (0.454)	**-0.023 **(**4.49 × 10^-24^**)	**0.135 **(**0.004**)	0.120 (0.005)
*T. gondii*	0.000 (0.998)	0.106 (0.315)	**-0.022 (1.29 × 10^-13^)**	0.120 (0.054)	**0.201 **(**6.61 × 10^-4^**)
CMV	-0.291 (0.072)	-0.181 (0.021)	**-0.031 (1.09 × 10^-38^)**	-0.011 (0.809)	0.020 (0.647)
EBV	**-0.624 (3.89 × 10^-4^)**	0.151 (0.066)	0.001 (0.693)	-0.002 (0.970)	0.034 (0.447)
HSV-1	**-0.798 (4.37 × 10^-5^)**	-0.008 (0.925)	**-0.009 (4.08 × 10^-4^)**	0.090 (0.084)	0.106 (0.029)
HSV-2	0.128 (0.350)	**-0.320 (2.19 × 10^-4^)**	**-0.018 (3.32 × 10^-13^)**	0.011 (0.822)	0.098 (0.033)
HHV-6	**0.481 (8.97 × 10^-4^)**	0.194 (0.065)	**0.021 (2.41 × 10^-13^)**	**-0.172(0.003)**	-0.118 (0.039)
VZV	0.230 (0.387)	0.150 (0.407)	0.000 (0.956)	-0.071 (0.519)	-0.181 (0.077)
Ad-36	-0.359 (0.011)	-0.048 (0.607)	0.000 (0.991)	0.023 (0.671)	0.131 (0.010)
HAV	**-1.143 (2.29 × 10^-7^**)	-0.024 (0.770)	**-0.038 (8.58 × 10^-46^)**	0.132 (0.014)	0.062 (0.179)
Influenza A	0.149 (0.412)	0.206 (0.045)	-0.005 (0.117)	-0.087 (0.154)	-0.083 (0.143)
Influenza B	**0.632 (4.52 × 10^-5^)**	0.128 (0.136)	0.003 (0.267)	-0.075 (0.147)	-0.014 (0.769)

All p-values in bold remain statistically significant after correcting for multiple testing across 13 pathogens using a conservative Sidàk correction.

* Analysis of location compares 147 individuals in Mexico with 1,227 participants in the U.S. All remaining analyses presented in this table refer only to the 1,227 U.S. participants. Positive regression coefficient refers to higher seroprevalence in the U.S. compared to Mexico (e.g., HHV-6 seroprevalence is higher for U.S. participants).

†Positive regression coefficient relates to a higher frequency in males compared to females (e.g., *C. pneumoniae* seroprevalence is higher in males).

Serostatus is a discrete trait, and since we used a liability threshold model for analysis of such dichotomous traits within a variance components model for analysis of family data [29], the direction of effect of the predictor on the pathogen is opposite of the sign of the regression coefficient (e.g., *H. pylori* seroprevalence decreases with higher education).

The overall seroprevalence estimates by sex and age category for each pathogen are presented in Table 2. The pathogen with the highest seroprevalence estimate in the Mexican American study population was VZV, at 92% (98% including the indeterminate samples among the seropositive ones). The overall seroprevalence rates of other herpesviruses were 82% for HSV-1, 82% for HHV-6, 55% for CMV, 46% for EBV, and 22% for HSV-2. The seroprevalence rates for the other viruses were 77% for influenza A, 58% for influenza B, 73% for HAV, and 13% for Ad-36. Of the bacterial pathogens, *C. pneumoniae* had a high seroprevalence rate (86%), and *H. pylori* had a seroprevalence of 57%. *T. gondii* displayed the lowest seroprevalence rate among the pathogens tested (9%). Although vaccines currently exist for influenza, VZV, and HAV, only influenza vaccines were available at the time of collection of blood specimens for this study (1991-1995).

For the majority of pathogens in this study, seroprevalence was similar between the sexes (Tables 2 and 3). However, a significant difference in seroprevalence was observed for HSV-2, which was higher in women (25%) than men (16%) (p = 2.2 × 10^-4^, after adjusting for age [Table 3]).

**Figure 1:**
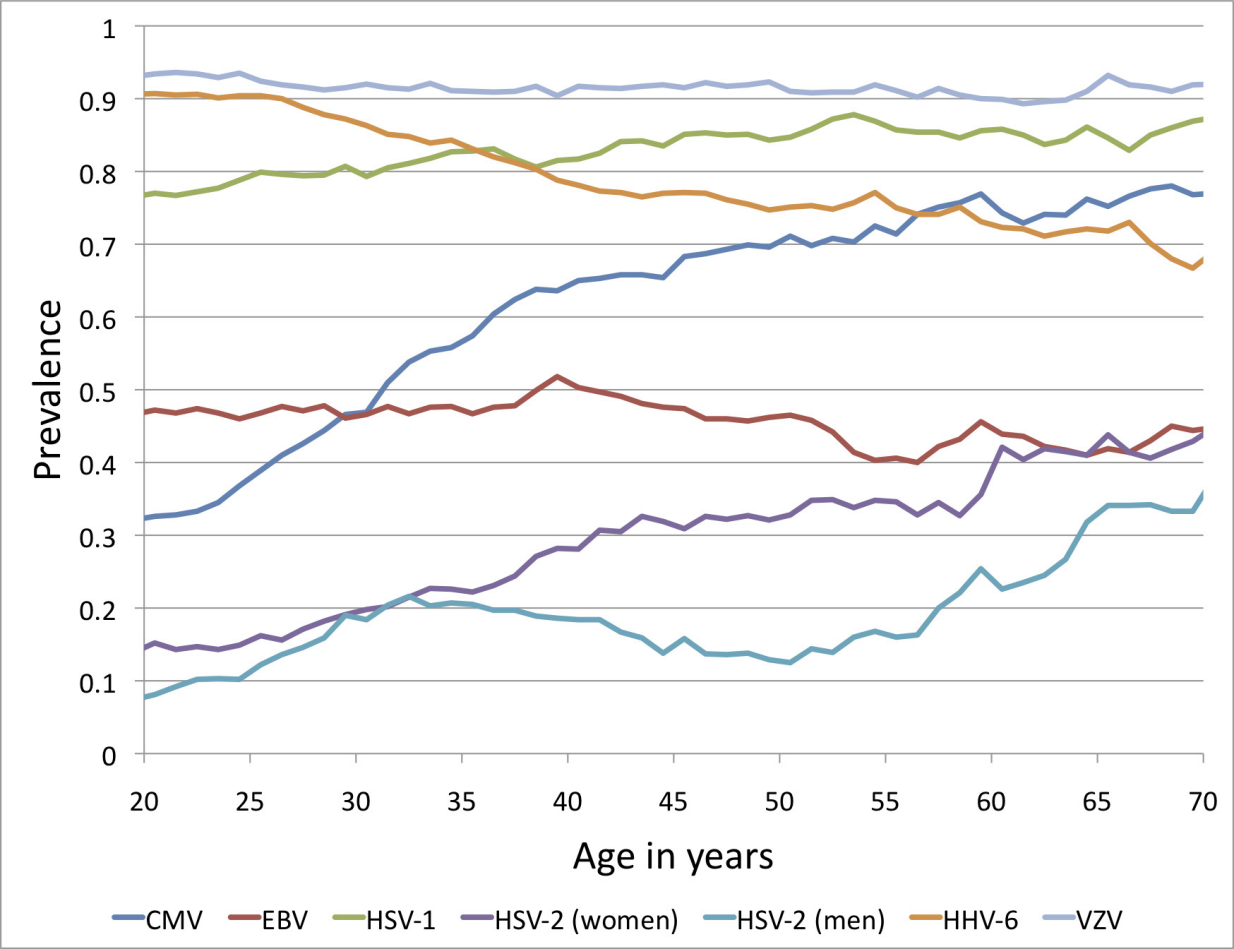
**Seroprevalence of human herpesviruses by age (sliding 15-year age windows used to smooth the curves, and age shown is the midpoint of each age interval)**.

**Figure 2:**
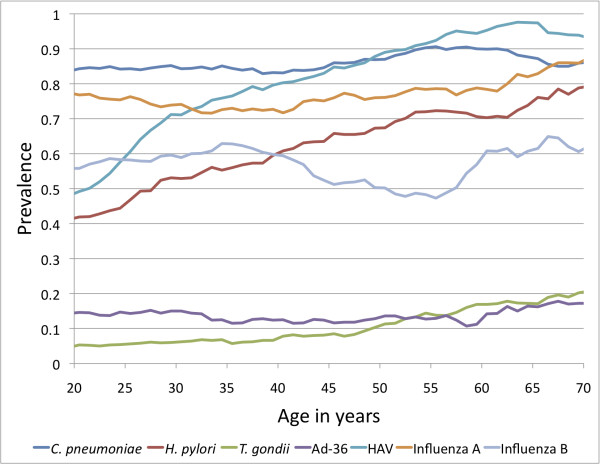
**Seroprevalence of miscellaneous pathogens by age (sliding 15-year age windows used to smooth the curves, and age shown is the midpoint of each age interval**.)

Figures 1 and 2 present the seroprevalence rates for all participants across the age categories, using sliding 15-year age windows to smooth the curves. Figure 1 includes the herpesviruses, and Figure 2 includes all other agents examined. Rates for males and females were combined for each pathogen, except for HSV-2. Most pathogens significantly increased in seroprevalence with age, as expected, since there would be a greater opportunity for exposure with age. For example, the rate of seropositivity for *H. pylori* increased from ~40% at age 20 years, to ~80% by the age of 70 years (p = 4.5 × 10^-24^). The seroprevalence of some of the pathogens, such as VZV, remained relatively stable across the age groups, with most individuals having acquired infection during childhood. HHV-6 is the only pathogen that showed a significant decrease in seroprevalence by age (p = 2.4 × 10^-13^). It is noteworthy that antibody concentrations for some pathogens tended to decline slightly in the oldest age groups, possibly due to the impact of aging on the immune system.

Education and income were also tested as potentially contributing to seroprevalence of the pathogens examined in this study (Table 3). After accounting for sex and age effects as well as multiple testing, a higher level of education was a significant predictor (p = 0.003) of an increased HHV-6 seropositivity rate, but a lower *H. pylori* seroprevalence (p = 0.004). Higher household income was significantly related to lower seroprevalence estimates of *T. gondii* (p = 6.6 × 10^-4^).

**Figure 3:**
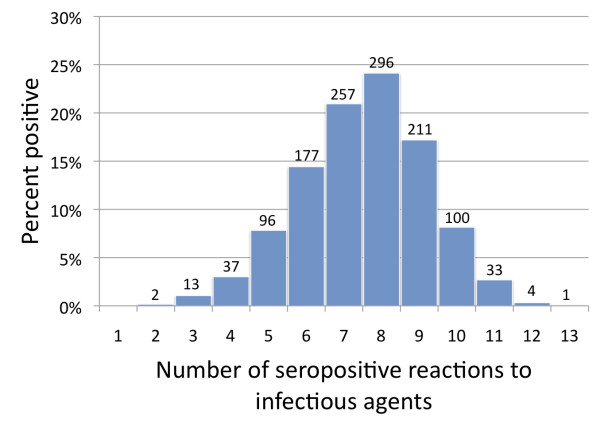
**Number of seropositive reactions to infectious agents for each study participant**.

We also assessed serological evidence of exposure to multiple pathogens. As demonstrated in Figure 3, all study participants were seroreactive to at least 2 of 13 pathogens examined. The majority of participants (77%) were seroreactive to 6 to 9 of the infections tested, with a single subject showing seropositivity to all 13 of the pathogens. On average, participants were seropositive to 8 of the pathogens examined.

## Discussion

Comparing seroprevalence across studies is fraught with difficulty because assays may vary, and modest changes in antibody titer thresholds for defining serostatus categories (positive, indeterminate, or negative) can have substantial impacts on the estimated seroprevalence. These issues are less of a concern for examining seroprevalence within a single study, which is why we have focused our examination on the influence of sex, age, and other factors within this Mexican American population. Nonetheless, a brief comparison to published seroprevalence data is provided (Table 4). Where possible, we used for comparison data derived from the National Health and Nutrition Examination Survey (NHANES) III, a nationally representative survey of the U.S. population that contains data on minority populations, including Mexican Americans, and covers the time period 1988 to 1994, which overlaps with this study (1991-1995). Although routine childhood vaccinations against two pathogens (VZV and HAV) were implemented in the U.S. in 1995, it does not affect the results of this study or NHANES III, as both sets of data were collected during the pre-vaccine period. While the data presented in this study may be considered "historical" in that they were collected 15- 20 years ago, they are crucial for investigating the relationship between infections due to common pathogens and chronic diseases of aging. In addition, they serve as a reference for studies investigating epidemiological changes that may have occurred over time. Although every population is different, in one of our own studies of Alaskan Natives [31] we found that seroprevalence rates, including for some of the same pathogens investigated here, have remained relatively stable over time in that population.

**Table 4:** Seroprevalence comparisons with published studies

Pathogens	Seroprevalence rates for this study, %	Seroprevalence rates for comparative studies, % (description of population)
*C. pneumoniae*	86	63 (Brooklyn, NY, USA) [32], 42 (Alaskan Natives) [31]
*H. pylori*	57	62 (Mexican American)* [62], 26 (non-Hispanic White)* [62]
*T. gondii*	9	25 (Mexican American)* [33], 24 (non-Hispanic White)* [33], 25 (general U.S. population)* [33]
CMV	55	85 (Mexican American)* [6], 57 (non-Hispanic White)* [6], 62 (general U.S. population)* [6]
EBV	46	73-90 (general U.S. adult population) [34]
HSV-1	82	88 (Mexican American)* [6], 65 (non-Hispanic White)* [6], 68 (general U.S. population)* [6]
HSV-2	22 (total) 25 (women) 16 (men)	38 (Mexican American women)* [33], 27 (Mexican American men)* [33], 65 (non-Hispanic Black women)* [33], 43 (non-Hispanic Black men)* [33], 22 (non-Hispanic White women)* [33], 18 (non-Hispanic White men)* [33], 25 (general U.S. population)* [33]
HHV-6	82	60-70 [63]; 66-94 [4]
VZV	92	97(Mexican American)* [64], 97 (non-Hispanic White)* [64], 96 (general U.S. population)* [64]
Ad-36	13	11 (non-obese) [36], 30 (obese) [36]
HAV	73	82 (Mexican American)* [33], 29 (non-Hispanic White)* [33]

* NHANES III data

Many of the pathogens quantified in this study exhibit seroprevalence estimates similar to published reports of Mexican Americans or the general U.S. population (Table 4). These include the herpesviruses HHV-6 and VZV. Pathogens with seroprevalence rates similar to other Mexican Americans, but higher than non-Hispanic Whites, include HAV, *H. pylori*, and HSV-1. CMV and HSV-2 seroprevalence rates are similar to previous data for non-Hispanic Whites and are lower than other Mexican American populations. *C. pneumoniae *seroprevalence in this study (86%) is elevated compared to most published literature reports (e.g., 63% for Brooklyn, NY) [32]. In contrast, *T. gondii* seroprevalence among study participants (9%) is substantially lower than that observed in Mexican Americans (25%) from one recent published study of NHANES III sera [33].

The seroprevalence of EBV reported in this study (minimum 47%, maximum 70% including indeterminates) is somewhat lower than seroprevalence rates reported in other adult populations [34]. It should be noted that this estimate was published in 1969, which is much earlier than the present study. In addition, other seroepidemiologic studies have typically measured IgG antibodies to EBV viral capsid antigen (VCA), whereas an EBNA IgG assay was utilized in our study. Although most EBV-infected adults are both EBNA IgG and VCA IgG seropositive, some persons do not generate an EBNA response, and others may lose antibody titers over time (especially immunocompromised populations) [35]. EBV seroprevalence may also be related to the particular anti-EBNA IgG ELISA test utilized for this study, and the cut-off values established. A seropositivity rate similar to ours, using the same EBNA ELISA assay, was recently noted in another adult U.S. population (R. Yolken, data not shown).

Data are accumulating from several groups from Italy, Korea and the U.S. for the possible role of Ad-36 in contributing to obesity in humans [36-39]; however, one U.S. study did not find an association of Ad-36 with human obesity [40]. In this first study to characterize Ad-36 seroprevalence in a Hispanic population, the 13% infection rate observed for Ad-36 in these subjects was lower than the 30% rate observed in obese non-Hispanic subjects reported earlier [36], despite substantial rates of obesity among study participants (39% obese, mean BMI of 29). It should also be noted, however, that there are differences in the timing of sample collection between the two studies, as the samples used in the other study were collected more recently.

The seroprevalence rates for influenza A and B presented in this study (77% and 58%, respectively) are based on whole virus assays that do not distinguish between naturally acquired antibodies and those resulting from seasonal vaccinations (which typically include two A and one B influenza virus strains). These data were not comparable to type-specific assays, and therefore were not included in the comparisons presented in Table 4.

Approximately half of the pathogens exhibited significant differences in sex- and age-adjusted seroprevalence by location of residence. Pathogens *C. pneumoniae*, EBV, HSV-1, and HAV were significantly higher in seroprevalence in subjects from Mexico. Several of these agents (e.g., *C. pneumoniae, H. pylori*, HSV-1, and HAV) are known to be associated with lower socioeconomic status, crowded conditions, or residence in a developing country [41-46,32]. Two pathogens, HHV-6 and influenza B, exhibited higher seroprevalence among individuals located in the U.S. HHV-6 seropositivity was also associated with higher education. Higher rates of influenza B may be due to increased seasonal flu vaccination rates among individuals residing in the U.S., or higher circulation of influenza B in the U.S. during preceding years.

The only pathogen in this study for which there was a statistically significant difference in seroprevalence rates between the sexes was HSV-2, at 25% in women and 16% in men. Higher rates of HSV-2 seroprevalence in women have been reported worldwide, as summarized previously [45], and are attributable to a higher risk of acquisition of this sexually transmitted pathogen in women.

The significant increase in seroprevalence rates by age for six of the 13 pathogens (*H. pylori, T. gondii,* CMV, HSV-1, HSV-2, and HAV) is likely the result of more opportunities for exposure as a person ages. For the herpesviruses this may also be due to a reactivation of the virus after a period of latency, and for *T. gondii,* this could occur from the activation of relatively slow growing organisms in tissue cysts. However, our observed increase in seroprevalence rates with age may also be due to a cohort effect wherein pathogen exposure was more frequent among individuals belonging to the older age groups. This is supported by evidence for a trend toward decreased HAV, HSV-1 and HSV-2 seroprevalence rates in the U.S., probably resulting from improved hygiene and sanitation, or changes in sexual practices [43,46]. Among participants of this study, HHV-6 was the only pathogen that decreased significantly with age. Previous studies indicate that HHV-6 seroprevalence rates peak in early childhood, at approximately three years of age, followed by a decline in antibody titer thereafter [47]. Among adults, continued decline in antibody titer with age indicates that HHV-6 reinfection or reactivation is rare [48].

In this study the effects of socioeconomic factors, including education and income, were assessed to determine their effect on pathogen serostatus. The enteric pathogen *H. pylori* showed a significant decline in seroprevalence rate associated with higher level of educational achievement, likely related to better hygiene and sanitation, and perhaps also to less household crowding and smaller family size. Seropositivity for HHV-6, on the other hand, increased significantly with more education. Given that this pathogen is typically acquired during early childhood, this higher seroprevalence rate may be related to activities promoting transmission that are utilized more frequently in those with higher education (e.g. day care). *T. gondii *exhibited a significant decline in seropositivity related to increased household income. Differences in culinary practices (e.g. avoidance of raw or undercooked meat) and/or hygiene (e.g. avoidance of food, water, and soil contaminated by shed oocytes from cat feces) likely contribute to lower *T. gondii *seroprevalence [49].

## Conclusions

This study examined the seroprevalence rates of 13 common infectious pathogens in a geographically defined group of Mexican Americans from San Antonio, Texas, who suffer from substantial rates of obesity, type 2 diabetes, cardiovascular risk factors, and other chronic disease [24,50-53]. The study population may also be at elevated risk for many infections associated with poor hygienic conditions and crowding, given the modest average socioeconomic status of the study participants. Our seroprevalence data for many of the pathogens is consistent with this hypothesis. Of the pathogens prevalent in this group, *H. pylori*, *C. pneumoniae*, HSV-1, HAV, and CMV have all been linked to chronic illness, including cardiovascular disease [54-59]. Studies also indicate that multiple infections may increase the risk of infection with additional pathogens, increase the severity of the other infections, or both [60,61,6]. The assessment of serological evidence of exposure to multiple pathogens demonstrates that the Mexican American participants in this study have serological evidence of exposure to a number of different pathogens, potentially influencing health and contributing to the development of chronic disease in this population from San Antonio, Texas.

## Competing interests

The authors declare that they have no competing interests.

## Authors' contributions

HHHG, CTL and JB conceived the study. JB provided plasma samples and relevant information on study participants. CTL, BG, RY, and ND performed or supervised the serological assays. RR performed the statistical analyses, with help from HHHG. RR and HHHG drafted the manuscript, with editing from CTL, EK, and RY. All authors read and approved the final manuscript.

## Supplementary Material

Additional File 1 **Seroprevalence estimates for participants residing in Mexico**. Table containing seroprevalence estimates by sex for the 13 pathogens examined in this study for 147 individuals residing in Mexico. Click here for file
