# Supplementary material for: Seroprevalence of 13 common pathogens in a rapidly growing U.S. minority population: Mexican Americans from San Antonio, TX
Source: BMC Res Notes. 2011 Oct 21;4:433. doi: 10.1186/1756-0500-4-433 (PMC3214184; doi:10.1186/1756-0500-4-433)
Supplement: Additional File 1 — Seroprevalence estimates for participants residing in Mexico. Table containing seroprevalence estimates by sex for the 13 pathogens examined in this study for 147 individuals residing in Mexico. [file 1756-0500-4-433-S1.DOC]

## Additional File 1: Table 1S - Seroprevalence estimates for participants residing in Mexico

|  | count | *Cp** | *Hp* | *Tg* | CMV | EBV* | HSV-1* | HSV-2 | HHV-6* | VZV | Ad-36 | HAV* | IA | IB* |
| --- | --- | --- | --- | --- | --- | --- | --- | --- | --- | --- | --- | --- | --- | --- |
| Females | 80 | 97.5 | 58.8 | 10.0 | 77.5 | 62.5 | 96.3 | 18.8 | 68.8 | 90.0 | 31.3 | 98.8 | 66.3 | 36.3 |
| Males | 67 | 100 | 62.7 | 11.9 | 59.7 | 58.2 | 95.5 | 20.9 | 65.7 | 95.5 | 11.9 | 94.0 | 82.1 | 32.8 |
| Overall  (including indeterminates) | 147 | 98.6  (98.6) | 60.5  (64.6) | 10.9  (12.9) | 69.4  (77.6) | 60.5  (85.7) | 95.9  (95.9) | 19.7  (21.1) | 67.3  (76.9) | 92.5  (97.3) | 22.4  (22.4) | 96.6  (96.6) | 73.5  (89.8) | 34.7  (59.9) |

Cp=C. pneumoniae; Hp=H.pylori; Tg=T. gondii; IA=Influenza A; IB=Influenza B

*Pathogens with seroprevalence rates that differ significantly between study participants residing in Mexico compared to the U.S.
